# Microbial and Plant-Assisted Bioremediation of Heavy Metal Polluted Environments: A Review

**DOI:** 10.3390/ijerph14121504

**Published:** 2017-12-04

**Authors:** Omena Bernard Ojuederie, Olubukola Oluranti Babalola

**Affiliations:** Food Security and Safety Niche Area, Faculty of Natural and Agricultural Sciences, North-West University, Private Mail Bag X2046, Mmabatho 2735, South Africa; 30783887@nwu.ac.za

**Keywords:** anthropogenic sources, environmental pollution, genetically engineered organisms, heavy metals, microbial remediation, phytoremediation

## Abstract

Environmental pollution from hazardous waste materials, organic pollutants and heavy metals, has adversely affected the natural ecosystem to the detriment of man. These pollutants arise from anthropogenic sources as well as natural disasters such as hurricanes and volcanic eruptions. Toxic metals could accumulate in agricultural soils and get into the food chain, thereby becoming a major threat to food security. Conventional and physical methods are expensive and not effective in areas with low metal toxicity. Bioremediation is therefore an eco-friendly and efficient method of reclaiming environments contaminated with heavy metals by making use of the inherent biological mechanisms of microorganisms and plants to eradicate hazardous contaminants. This review discusses the toxic effects of heavy metal pollution and the mechanisms used by microbes and plants for environmental remediation. It also emphasized the importance of modern biotechnological techniques and approaches in improving the ability of microbial enzymes to effectively degrade heavy metals at a faster rate, highlighting recent advances in microbial bioremediation and phytoremediation for the removal of heavy metals from the environment as well as future prospects and limitations. However, strict adherence to biosafety regulations must be followed in the use of biotechnological methods to ensure safety of the environment.

## 1. Introduction

Pollution of the environment keeps on increasing at an alarming rate due to the activities of man such as urbanization, technological advancement, unsafe agricultural practices and rapid industrialization which degrades the environment. Heavy metals released into the environment are persistent due to their toxicity which poses a severe threat to organisms exposed to high levels of such pollutants. Metals are essential to the biological functions of plants and animals but at elevated levels, they interfere with metabolic reactions in systems of organisms. Toxic heavy metals such as lead (Pb), cadmium (Cd), mercury (Hg), chromium (Cr), zinc (Zn), uranium (Ur), selenium (Se), silver (Ag), gold (Au), nickel (Ni) and arsenic (As) which are not useful to plants, are capable of reducing plant growth due to reduced photosynthetic activities, plant mineral nutrition, and reduced activity of essential enzymes [1,2]. Heavy metals are cytotoxic at low concentrations and could lead to cancer in humans [3]. These toxic metals could accumulate in the body when consumed in contaminated food through the food chain and become health risks to living organisms [4]. This causes oxidative stress, an unevenness involving the production of free radicals and the capacity of cells to eradicate them or repair the damage [5,6]. This leads to base damage through formation of reactive oxygen species (ROS) which includes oxygen radicals (superoxide and hydroxyl) [7] and non-radical derivatives of molecular oxygen (O_2_) such as hydrogen peroxide (H_2_O_2_), as well as breakage of the DNA molecule [5,6]. Heavy metal toxicity increases the production of ROS thereby decreasing the antioxidant systems (glutathione, superoxide dismutase, etc.) which protect cells. If this condition continues, the normal functioning of the organism is affected and may invariably lead to cell death.

Bioremediation is gradually being accepted as the standard practice for the restoration of heavy-metal-contaminated soils since it is more eco-friendly and cost effective compared to the conventional chemical and physical methods, which are often very expensive and ineffective when metal concentrations are low, in addition to producing significant amounts of toxic sludge [8,9]. The cost effectiveness of bioremediation was reported by Blaylock et al. [10] who were able to save 50–65% of cost, when bioremediation was used for the treatment of one acre of Pb-polluted soil compared with the use of conventional methods such as excavation and landfill [7]. The ability of microorganisms to degrade pollutants depends on the suitability of environmental conditions for their growth and metabolism which include suitable temperature, pH, and moisture [11]. This review discusses the effects of heavy metals on the environment and how they can be effectively remediated using plants and microorganism. It further discusses the various mechanisms utilized by these organisms for remediating heavy metal contamination. The possible prospects and limitation of genetically modified organisms for bioremediation are also discussed.

## 2. Bioremediation

Bioremediation is a technique used to remove environmental contaminants from the ecosystem. It utilizes the biological mechanisms inherent in microbes and plants to eradicate hazardous pollutants and restore the ecosystem to its original condition [9]. The basic principles of bioremediation involve reducing the solubility of these environmental contaminants by changing pH, the redox reactions and adsorption of contaminants from polluted environment [12]. Various reports have been made on enhancing biosorption of pentachlorophenol (PCP) by altering the pH levels in aqueous solutions. For example, the biosorption abilities of *Aspergillus niger* [13] and *Mycobacterium chlorophenolicum* [14,15] in the removal of PCP from aqueous solutions were reported to be pH-dependent. Brandt et al. [14] also evaluated the influence of pH on adsorption and desorption behavior of PCP by *M. chlorophenolicum* and reported that pH values were an essential parameter which affected PCP adsorption, with adsorptive capacity increasing with decreased pH. At pH 5.4, adsorption by the bacterium was completely irreversible, while complete desorption was obtained at pH 7.0. At pH 6–8, better results on adsorption behavior of PCP by microbial biomass in aqueous solution were obtained by Jianlong et al. [16]. The results obtained by various authors highlight the importance of using the appropriate pH for optimum performance of microorganisms used in bioremediation. Bioremediation technologies are based on redox processes which focus on modifying the chemistry and microbiology of water by injecting selected reagents into contaminated water to enhance the degradation and extraction of various contaminants by in situ chemical oxidation/reduction reactions [17,18]. Redox reactions involve chemically transforming harmful contaminants into innocuous or less toxic compounds that are more stable, less mobile or inert [18]. It plays a vital role in the transformation of toxic heavy metals, especially As, Cr, Hg and Se in soils and sediments into less toxic or innocuous forms [19,20]. Redox reactions in contaminated soil sediments and groundwater are often affected by the physicochemical properties of the medium, but this can be manipulated by addition of organic and inorganic amendments such as composts and biochar [21,22]. The application of organic amendments such as compost in metal-contaminated soils could cause differences in the soil microbial population by changing pH, decreasing the solubility of heavy metals and increasing allochthonous microbial biomass and available nutrients [23,24]. Biochar is a product of pyrolysis of biomass obtained from sources such as crop residue, manure and solid wastes which can be used to stimulate microorganisms for bioremediation by making the environment more favorable [25]. Comprehensive reviews by several authors have described the potential value of biochar as an effective agent in immobilization of metals and organic pollutants [26,27,28,29]. Biochar has the ability to donate, accept or transfer electrons within their environments abioticaly or through biological pathways [30,31]. It has been suggested by some researchers that biochar may also facilitate microbial electron shuttling processes since they display similar functional characteristics to soil redox-active organic matter [29,32]. Biochar acts by increasing the pH of contaminated soils thereby affecting the bioavailability of heavy metals for plant uptake. The mobility and toxicity of many elements, such as chromium, selenium, lead, arsenic, nickel and copper, rely basically on their oxidation states which, in turn, are controlled by the redox reactions [18,33]. Tandon et al. [34] reported a new oxidative route for transformation of As (III) to As (V) using clay-supported zerovalent iron nanoparticles by mixing ferric nitrate with liquor of commercially available tea. Up to 99% removal of As (III) from contaminated water was achieved.

The effectiveness of bioremediation depends on several factors such as the nature of the organisms utilized, the prevailing environmental factors at the contaminated site, as well as the degree of the pollutants in that environment [35]. Bioremediation can be achieved with the use of microorganisms (microbial bioremediation) which depends on the metabolic potential of the microorganisms to degrade environmental pollutants and change them to innocuous forms through redox processes [36]. It can also be carried out by plants which bind, extract and remediate pollutants from the environment (phytoremediation). The level of contaminated soil, the bioavailability of the metal contaminant, as well as the accumulation of metals as biomass by the plant, are critical to the success of phytoremediation as a means of eradicating heavy metals from contaminated sites using plants [4]. Bioremediation could be in-situ or ex-situ. In-situ bioremediation is an onsite cleanup process of contaminated environments which involves supplementing contaminated soils with nutrients to stimulate microorganisms in their ability to degrade contaminants, as well as add new microorganisms to the environment or improve the indigenous microorganisms to degrade specific contaminants using genetic engineering [37,38]. Utilization of natural microorganisms in the environment for in situ bioremediation is affected by the non-availability of suitable nutrient levels and/or environmental setting at the polluted location [39,40]. Ex-situ bioremediation involves taking the contaminated media from its original site to a different location for treatment based on the cost of treatment, deepness of contamination, pollutant type and the extent of pollution, geographical locality and geology of the contaminated site [35].

## 3. Effects of Heavy Metals on the Environment

The non-biodegradability of heavy metals makes it hard to remove them from contaminated biological tissues and this is a major concern for global health because of their lethal nature [9]. Heavy metals such as cobalt (Co), copper (Cu), iron (Fe), manganese (Mn) and molybdenum (Mo) are required in small quantities for the survival of living organisms [41], but at higher concentrations, they could become detrimental. The heavy metals Hg, Cr, As, Zn, Cd, Ur, Se, Ag, Au and Ni are hazardous heavy metals that contaminate the environment and adversely affects the quality of the soil, crop production as well as public health [9,42,43,44,45,46], if their concentration exceeds the maximum permissible concentration in water given by the Comprehensive Environmental Response Compensation and Liability Act, USA: Ar (0.01 mg·L^−1^), Cd (0.05 mg·L^−1^), Cr (0.01 mg·L^−1^), Pb (0.015 mg·L^−1^), Hg (0.002 mg·L^−1^) and Ag (0.05 mg·L^−1^) respectively [41,47]. These pollutants are major sources of life-threatening degenerative diseases affecting humans such as cancer, Alzheimer’s disease, atherosclerosis, Parkinson’s disease, etc. [48]. The degree of toxicity of each metal is determined by the duration of exposure as well as the absorbed dosage by the organisms. Among organisms greatly affected by heavy metal toxicity are plants as their normal physiological activities are severely hampered. For example, the processes of respiration, photosynthesis, electron transport chain and cell division are negatively affected by elevated levels of heavy metals as documented by laboratory experiments [49,50]. Moreover, high metal toxicity inhibits cytoplasmic enzymes in plant cells and causes damage to cell structures due to oxidative stress [7,51] which consequently affects plant growth and metabolism. Exposure of the body to high levels of Pb could cause serious health implications such as lack of coordination and paralysis, while severe exposure to Cd damages internal organs of the body such as the kidney, liver and cardiac tissues [52]. Arsenic is the most common cause of acute heavy metal poisoning in adults and children [53,54] and could result in respiratory diseases such as reduced pulmonary function or lung cancer. The central nervous system is affected by Hg, a neurotoxin which impairs speech and hearing, and causes weakness of the muscles [55]. It accumulates in the cells of microbes in aquatic bodies where it gets converted to methyl mercury in the microbes and becomes detrimental to aquatic lives. Consumption of fish and other aquatic animals by man can cause the transfer of toxic methyl mercury to man. Due to the detrimental effects of these heavy metals, concerted efforts need to be made to effectively eradicate them from the environment and stabilize the ecosystem.

## 4. Mechanism of Heavy Metal Remediation by Microorganisms

Heavy metals are known to dislodge important components in biological molecules, hindering the functions of the molecules and changing enzyme, protein or membrane transporter structure or function thereby becoming toxic to plants [4]. The major treatment regimes used for heavy metal degradation include methods such as coagulation, chemical precipitation, electrodialysis, evaporative recovery, floatation, flocculation, ion exchange, nanofilteration, reverse osmosis, ultrafiltration, etc. [56], as well as physico-chemical methods such as extraction, stabilization, immobilization, soil washing, etc. These methods, even if effective, are generally expensive as a result of high energy and chemical reagent requirements, apart from production of secondary noxious end-products [57]. An efficient way of removing toxic metal contaminants from the environment and stabilizing the ecosystem is to make use of indigenous microorganism with mechanisms capable of degrading such heavy metals, or genetically engineered microorganisms to treat polluted environments by converting toxic heavy metals into non-hazardous forms [42]. However, the bioremediation process will only be successful if only microorganisms with proven ability to remediate and tolerate heavy toxicity are utilized.

Microorganisms are essential in remediation of heavy-metal-contaminated environments as they have a variety of ways to endure metal toxicity. The exploitation of microorganisms to sequester, precipitate, or change the oxidation state of numerous heavy metals has been widely studied [42,58]. Bioremediation of heavy metals will be successful if a consortia of bacterial strains is utilized rather than using a single strain culture. In the study of Kang et al. [59], the synergistic effect of bacterial mixtures on the bioremediation of a mixture of Pb, Cd and Cu from contaminated soils using four strains: *Viridibacillus arenosi* B-21, *Sporosarcina soli* B-22, *Enterobacter* cloacae KJ-46 and *E. cloacae* KJ-47 was investigated. They observed that the bacterial mixtures had greater resistance and efficiency for the remediation of heavy metals compared to using single strain culture after 48 h with remediation efficiencies of 98.3% for Pb, 85.4% for Cd and 5.6% for Cu recorded. The following mechanisms are used for microbial bioremediation:

(1) Sequestration of toxic metals by cell wall components or by intracellular metal binding proteins and peptides such as metallothioneins (MT) and phytochelatins along with compounds such as bacterial siderophores which are mostly catecholates, compared to fungi that produce hydroxamate siderophores.
(2) Alteration of biochemical pathways to block metal uptake.
(3) Conversion of metals to innocuous forms by enzymes.
(4) Reduction of intracellular concentration of metals using precise efflux systems [36].

**Figure 1:**
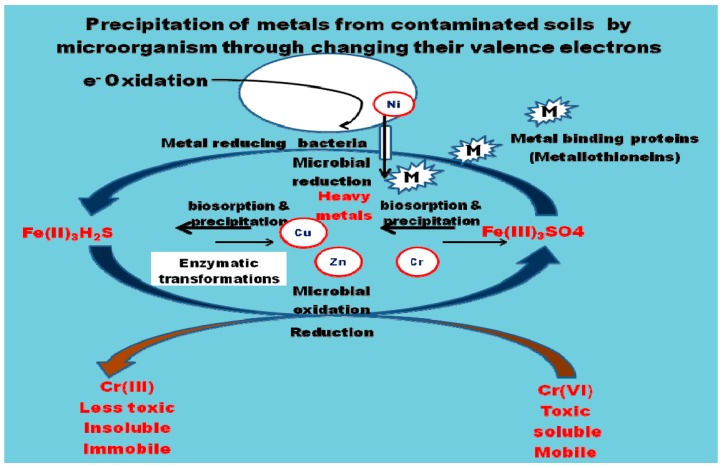
Mechanisms of removal of heavy metals from contaminated soils by microorganims through the processes of precipitation, biosorption via sequestration by intracellular metal binding proteins (metallothioneins) and conversion of metals to innocous forms by enzymes (enzymatic transformation).

The mechanisms used in remediation of heavy metals from contaminated soils are presented in Figure 1.

**Figure 2:**
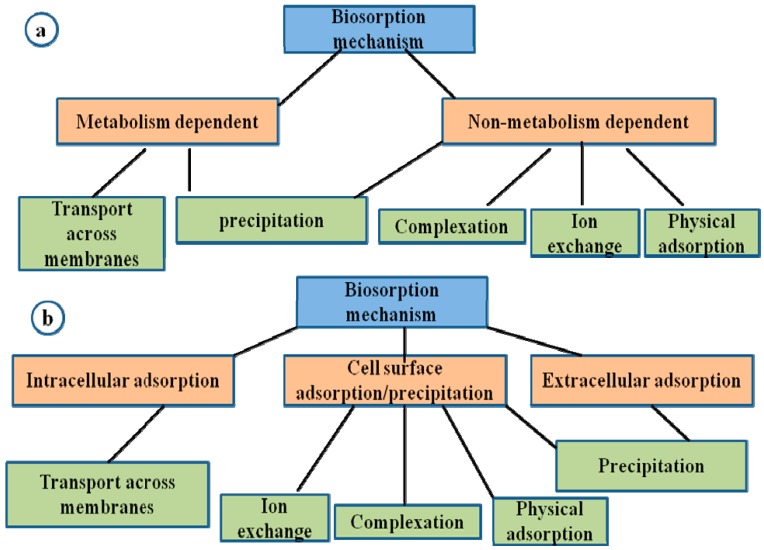
Mechanisms of biosorption based on (**a**) dependence on cell metabolism; (**b**) location within the cell where the metal is removed, adapted from Gupta et al. [43].

Bacteria produce iron-chelating substances called siderophores which enhances mobility and reduces bioavailability of metals and its subsequent removal from soil. Sulphate-reducing bacteria such as *Desulfovibrio desulfuricans* have the ability to convert sulphate to hydrogen sulphate which then reacts with heavy metals such as Cd and Zn to form insoluble forms of these metal sulphides [7]. Biosorption is the removal of heavy metals, compounds and particulates from a solution by low cost biological materials such as dead mass or natural materials with greater degradative ability [60,61]. The mechanisms involved in biosorption could either be dependent on the cell’s metabolism or the area of metal removal which is an independent metabolism. This could be extracellular accumulation/precipitation, cell surface sorption/precipitation and intracellular accumulation [22,23] as presented in Figure 2.

Biosorptive abilities of microbial biomass vary within groups but the biosorption competence of each biosorbent is affected by prehistory and pretreatment, as well as the experimental conditions [9]. The negatively charged functional groups present in biomolecules of microbial cell wall surfaces such as hydroxyl groups, phosphate groups, carbonyl groups, etc. bind readily to heavy metal ions [3,61]. Bacterial functional groups, such as uronic acid of carboxyl groups (RCOO^−^) and sulfate groups (SO_4_^2−^), are also capable of carrying out ion exchange [61]. Gram-positive bacteria cell walls consist of peptidoglycan layers that contain the amino acids alanine and glutamic acid as well as meso-di-aminopimelic acid and teichoic acid, while enzymes, glycoproteins, lipopolysaccharides, lipoproteins and phospholipids are present in Gram-negative bacteria cell walls [9]. These components of the cell wall are the active sites for binding processes in bacteria [62] as they act as ligands for binding metal ions, resulting in their ultimate remediation from contaminated environments [63]. Bacteria are essential biosorbents for the treatment of polluted environments because they are able to grow under controlled conditions and can withstand intense environmental conditions [64]. They act as good biosorbents for heavy metals from polluted environments. Likewise, fungi are able to withstand and detoxify metal ions by active accumulation, intracellular and extracellular precipitation, and valence transformation, hence they are potential biocatalysts for the bioremediation of heavy metals as they are able to absorb heavy metals into their mycelium and spores [9]. Yeast (*Sacharomyces cerevisiae*) are also used as efficient agents of bioremediation because they have the ability to remediate toxic metals from contaminated wastewaters by biosorption through the mechanism of ion exchange [61,65,66]. Algae turns out large biomass which gives them a high sorption capacity compared to other microbial biosorbents [9,67]. Mustapha and Halimoon [68] obtained biosorption efficiency of 15.3–84.6% using algae which is high compared to other microbial biosorbents. This takes place by ion exchange mechanisms. It was also reported that brown marine algae effectively remediate heavy metal such as Cd, Ni and Pb by chemical groups on their surfaces such as carboxyl, sulfonate, amino and sulfhydryl groups [68]. A summary of remediation of heavy metal contaminants using micororganisms is presented in Table 1.

Microbes make use of heavy metals and trace elements as terminal electron acceptors from which they acquire the needed energy to detoxify metals via enzymatic and non-enzymatic processes [3]. Bacterial cells are also capable of bioaccumulation which is the ability to build up heavy metal ions in both particulate as well as insoluble forms and their by-products. The most essential constituent in such bacterial cells having ion sequestration capability is exopolysaccharide (EPS). Exopolysaccharide is mainly composed of complex high molecular weight organic macromolecules like polysaccharide along with smaller proportions of protein and uronic acid [56]. Exopolysaccharide protects the bacteria against environmental stresses such as heavy metal toxicity, drought, salinity, etc. Microorganisms such as *Agrobacterium* spp., *Alcaligenes faecalis*, *Xanthomonas campestris*, *Bacillus* spp., *Zygomonas mobilis*, *Leuconostoc*, *Pseudomonas* spp. and *Acetobacter xylinum*, have been identified as genera of EPS-producing microorganisms [69]. The strategies for achieving heavy metal remediation through bacterial EPS has to be focused on utilizing the non neutral, negatively charged EPS (EPS packed with abundant anionic functional groups) to be incorporated as a suitable biosorbent [70]. Some commercial bacterial EPS with the required anionicity includes; alginate (*Pseudomonas aeruginosa*, *Azotobacter vinelandii*), gellan (*Sphingomonas paucimobilis*), hyaluronan (*Pseudomonas aeruginosa*, *Pasteurella multocida*, *Streptococci* attenuated strains), xanthan (*Xanthomonas campestris*), galactopol (*Pseudomonas oleovorans*) and fucopol (*Enterobacter* A47) [56]. Exopolysaccharide production is associated with processes such as biofilm production which is essential in the biosorption and biomineralization of metal ions [71].

Biofilms constitute a consortia of microorganisms enclosed in an extracellular matrix of polysaccharides, exudates and detritu even though they can also be made by a single bacterial species [86,87]. Exopolysaccharide can be modified chemically by acetylation, carboxymethylation, methylation, phosphorylation and sulphonylation which modifies the biological activities of EPS, thereby enhancing the applicability of the polymer [56]. More EPS-producing microorganisms need to be investigated for metal-ion-chelation abilities as they might come up with potent polysaccharide rich in anionic groups which will enhance the cleanup of the envionment from toxic metals.

## 5. Phytoremediation

**Figure 3:**
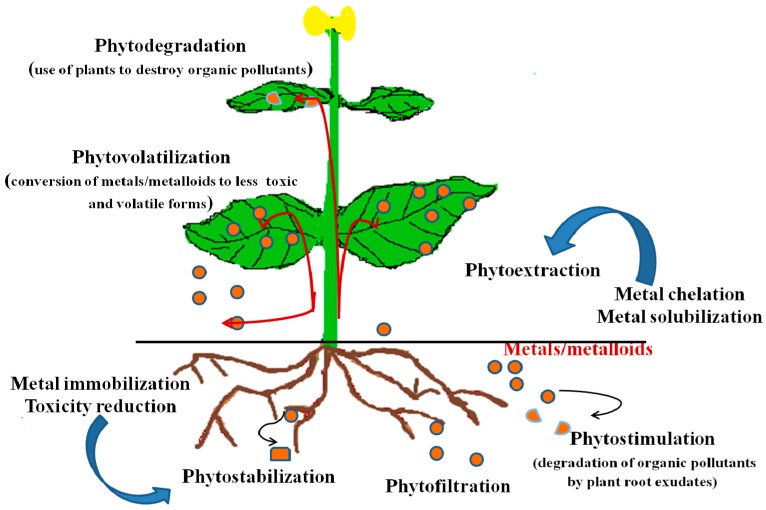
Processes used in phytoremediation of heavy metals.

Phytoremediation deals with the cleanup of organic pollutants and heavy metal contaminants using plants and rhizospheric microorganisms [3,88,89]. It is inexpensive, eco-friendly and an efficient means of restoration of polluted environments especially those that of heavy metals. Nonetheless, the level of soil contamination, the quantity of metal contaminant in the soil, as well as the ability of plants to aggressively take up metals from the soil, determine the success of phytoremediation at any polluted site [4]. Plants utilized in phytoremediation are the hyperaccumulators with a very high heavy metal accumulation potential and little biomass efficiency, and non-hyperaccumulators which possess lesser extraction capacity than hyperaccumulators, but whose total biomass yield is substantially higher and are fast-growing species [90,91]. Several processes are used to remove heavy metals from contaminted soils by some plants as illustrated in Figure 3.

### 5.1. Phytoextraction/Phytoaccumulation

Phytoextraction involves the uptake and movement of metal pollutants in the soil through plant roots into above-ground components of the plants, based on the mechanism of hyperaccumulation [92]. Hyperaccumulator plants take up metals in large quantities from contaminated soils, then transport and accumulate them in organs above the ground at concentrations from 100 to 1000 times higher than those found in non-hyperaccumulating species without suffering any apparent phytotoxic effect [93,94], hence they are very suitable for phytoremediation. These plants are usually found growing in areas with long-lasting metal contamination in soil over time and produce abundant biomass that can be easily harvested. Van der Ent [95] gave the following criteria for hyperaccumulator plants based on metal concentrations in dried foliage (Cd 100, Co, Cu, Cr 300, Pb, Ni 1000, Zn 3000 and Mn (µg/g respectively)). Based on these criteria, about 500 taxa have been identified as hyperaccumulators of some metals and the popular ones are representatives of the following families: *Brassicaceae*, *Caryophylaceae*, *Violaceae*, *Fabaceae*, *Euphorbiaceae*, *Lamiaceae*, *Asteraceae*, *Cyperaceae*, *Poaceae*, *Cunouniaceae* and *Flacourtiaceae* [48], as indicated in Table 2. These plants are unique because of the following characteristics: (1) a much greater capacity to take up heavy metals from the soil; (2) enhanced root-to-shoot translocation of metal ions; (3) a much greater ability to detoxify and sequester extremely large amounts of heavy metals in the shoots [48,94]; (4) ability to grow fast; and (5) a profuse root system [93].

In recent times, much interest has been placed on the use of sunflower (*Helianthus annuus*) for phytoremediation of organic pollutants and heavy metals due to the plant’s ability to take up heavy metals from the environment. The site of accumulation of these heavy metals differs from one plant to another. Some authors have reported accumulation of heavy metals mainly in the roots of sunflower with little movement from the roots to the above ground mass [96,97,98], while others reported effective movement from the roots to above ground mass [99,100]. Recent studies by Angelova et al. [101] showed that distribution of heavy metals in organs of sunflower is selective for each metal since 59% Pb accumulated in the leaves and as low as 1% accumulated in the seeds. Similar observations were made for Zn and Cd which accumulated 47% and 79% in the leaves of sunflower, respectively. Hyperaccumulator plants such as certain species within the *Brassica* genus (*Brassica napus*, *Brassica juncea* and *Brassica rapa*) are fast growers with high biomass [102]. An ornamental hyperaccumulator plant *Micranthemum umbrosum* (J.F. Gmel.) S.F. Blake was able to remove a higher percentage of As (79.3–89.5% from 0 to 1.0 µg∙mL^−1^) compared to Cd (60–73.1% 0.3 to 30.0 µg∙mL^−1^ Cd) [103]. The results obtained by Islam et al. [103] revealed the efficiency of *M. umbrosum* as an efficient accumulator of Cd and a hyper-accumulator of As toxicity. Despite the benefits of phytoextraction, its effectiveness can be hampered if the heavy metal conentration is very high, few biomass is produced by the plant or its growth rate is slow, which will hinder metal uptake. In such cases the phytoextraction process can be enhanced by using chelators such as citric acid and EDTA which increase mobility of soil heavy metals [104,105], or the use of organic supplements such as chicken manure which has been proven to increase growth of the species *Rorippa globosa* and decreased soil-extractable Cd and concentrations of Cd [105,106]. The type of soil present at a polluted site and the degree of metal contamination determines the rate at which hyperaccumulating plants can remediate that site. Therefore, research must be designed to identify hyperaccumulating plants that are fast-growing with the ability to accumulate abundant biomass and be tolerant to several metals.

Research towards identification of hyperaccumulating plants should focus on evaluation of the effect of metal stress on beneficial microorganisms within the rhizosphere and crops, and developing better applications of bioremediation technologies for remediating metals from contaminated soils [4]. These suggestions made by Tak et al. [4] are indispensable as they will give researchers the ability to use the appropriate hyperaccumulating plants to obtain the best results in phytoremediation of polluted environments. The efficiency of phytoextraction is based on several factors which include: (a) the choice of plant used, (b) the degree of plant tolerance to higher concentrations of heavy metals and (c) the capacity of plants to drastically take up heavy metals and move them from the roots to exposed surfaces which are essential for the phytoextraction process [92]. Phytoextraction can be commercially viable; besides removal of heavy metals from the soil, it also produces biomass with added value [101,107]. Phytoextraction is the most preferred method used by plants for remediation of polluted environments as it is enhanced by plant growth promoting rhizobacteria (PGPR) associated with the plant roots.

### 5.2. Phytofiltration

Phytofiltration could be in any of the three forms of rhizofiltration (the use of plant roots), blastofiltration (the use of seedlings) and caulofiltration (the use of excised plant shoots) [3,123,124]. It is the cleanup of polluted environments using plant roots or seedlings from aqueous wastes. For the effective use of phytofiltration as a phytoremediation technique, more studies have to be carried out to identify the parts of the plant that would be more efficient in accumulating the metal contaminants. This is essential for the use of this technique in bioremediation.

### 5.3. Phytostimulation

Phytostimulation is the enhancement of microbial activity to degrade organic contaminants by exudates from plant roots. Ethylene at low concentration stimulates root elongation but at high levels inhibits cell division and DNA synthesis. Nevertheless, this can be prevented by reducing ethylene concentration in plants using the enzyme 1-aminocyclopropane-1-carboxylase deaminase which reduces abiotic stress in plants by balancing plant ethylene-level production [69,125]. This enzyme is made by PGPR linked with plant roots using exudates released by plants as carbon and energy sources to degrade metal contaminants [4].

### 5.4. Phytostabilization

This involves the use of plant roots to absorb pollutants from the soil and retain them within the rhizosphere, and get separated and stabilized, rendering them harmless and preventing the pollutants from spreading in the environment [126]. The accessibility or mobility of heavy metals in the environment is reduced by precipitation in the region around plant roots, root sorption, metal valence reduction and complexation [90,104,127,128]. The amount of metal in rhizosphere soil available for uptake determines how efficient metals are moved within the plant and the success of phytostabilization process [90,129,130]. Plants used in phytostabilization should have a broad root system and low mobility of metals from roots to shoots [103]. The phytostabilization ability of a plant could be enhanced by changing the pH and organic matter content by addition of biochar or compost which will increase plant yield and immobilize the metals. Phytostabilization is a better alternative of capturing metals in situ because the pollutants are not taken up into tissues of the plants and do not spread into the environment [4]. It focuses primarily on heavy metal sequestration only within the rhizosphere.

### 5.5. Phytovolatilization

This deals with the removal of soil contaminants by plants which are readily changed into vapour and consequently released into the atmosphere [88,124]. Tobacco plants have the ability to accumulate highly toxic methyl mercury from Hg-contaminated sites and transform it to the less toxic elemental Hg in a volatile form that escapes through the leaves to the atmosphere [37,131]. The conversion of contaminants into volatile forms released during phytovolatilization is due to the metabolic potentials of the plants in union with microorganisms residing inside the rhizosphere [4].

### 5.6. Phytodegradation

Phytodegradation is the breakdown of organic contaminants into non-hazardous forms by plant enzymes [88]. Specific enzymes such as nitroreductases and dehalogenases are used by plants to degrade organic contaminants [132]. These enzymes must be used at optimal conditions of temperature and pH for efficient degradation of contaminants. The degradation of organic pollutants in the soil could also be enhanced by rhizospheric microorganisms through the process of rhizodegradation [88,131,133,134]. This is made possible because the rhizospheric region of the plant contains elevated levels of nutrients released from the roots that draw more bacteria to aid degradation of the contaminants compared to bulk soil which has less organic compounds and, hence, would contain less microbes [135]. This process is, however, restricted only to removal of organic pollutants since heavy metals are nonbiodegradable.

### 5.7. Rhizofiltration

Rhizofiltration involves the elimination of toxic substances or pollutants from ground water through filtration by the roots of plants. The process of rhizofiltration is based on the mechanism of rhizospheric accumulation by plants. Terrestrial plants are more efficient for rhizofiltration compared to aquatic plants because they employ natural solar driven pumps to take up particular elements from the environment [136]. Plants that have the ability to take up and resist high concentrations of toxic metals such as hyperaccumulators are best suited for rhizofiltration. Introduction of PGPR to a contaminated site decreases metal toxicity in plants as bioavailability of such metals reduces, thereby increasing the capacity of plants to get rid of heavy metal contaminants and get protected from environmental stress [4]. Nevertheless, phytoremediation technology has some limitations which include: decrease in the rate at which remediation occurs which frequently becomes insufficient when there are many pollutants at the contaminated site, and the accumulation and storage of pollutants in the plant materials [129].

## 6. Plant Mechanisms for Metal Detoxification

Heavy metals at harmful levels obstruct normal plant functioning and act as an obstacle to metabolic processes in various ways, including dislodgment of amino acids which arise from the construction of bonds connecting heavy metals and sulfhydryl groups [137,138]. The functional groups of important molecules in the cell are hindered by metal toxicity and the normal functioning of enzymes and pigments in the disrupted biomolecules, which interferes with the structure of the cytoplasmic membrane [63,88,138], and consistently suppress photosynthesis, respiration and enzymatic activities [137,138,139]. Physico-chemical properties can be used to place bioactive metals into two groups; redox-active metals (Cr, Cu, Mn and Fe) and non-redox active metals (Cd, Ni, Hg, Zn and Al) [140]. Redox active metals directly generate oxidative stress in plants which disrupt cell homeostasis, affects DNA structure and function, causes damage to the chloroplast and accessory pigments and eventually the cell is destroyed by the production of ROS [137,141,142]. Alternatively, oxidative stress is generated indirectly by non-redox active metals by several mechanisms which restrain antioxidative enzymes, or induces ROS-producing enzymes [137,143].

Active antioxidant systems occur naturally in plants which remove the toxicity produced by ROS [44]. The first line of defense in opposition to heavy metals in plants is the use of physical barriers such as morphological structures like thick cuticle, biologically active tissues like trichomes and cell walls, as well as mycorrhizal symbiosis that can act as biophysical barriers when plants are under heavy metal stress [137]. If these metal ions surpass these barriers and enter tissues and cells of the plant, several cellular defense mechanisms are initiated by the plant to restrain and attenuate the detrimental effects of the heavy metal [144]. Plant cells lessen the undesirable effects of free radicals by generating enzymatic antioxidants such as superoxide dismutase, catalase and glutathione reductase, and non-enzymatic antioxidants such as ascorbate, glutathione, alkaloids, tocopherols, etc. [142,145], that remove the free radicals [146]. Defense mechanisms used by plants include: production of the enzyme phytochelatin synthase that readily binds to heavy metals at lethal levels [89,147,148], production of metallothioneins [89,149] and production of proline that acts as a compatible and metabolic osmolyte, a component of cell walls, free radical scavenger, antioxidant and macromolecule stabilizer [150].

Phytochelatins (PCs) are short-chain thiol-rich repetitions of peptides of low-molecular weight synthesized from sulfur-rich glutathione by the enzyme phytochelatin synthase which act as defensive mechanism of plants against environmental stresses such as salinity, drought, herbicide and heavy metals [137]. They are used as biomarkers for the early detection of heavy metal stress in plants [151]. Plant metallothioneins (MTs) are cysteine-rich, low-molecular-weight and metal-binding proteins, synthesized due to mRNA translation [152,153]. They have much affinity for a wide range of metals such as Cu, Zn, Cd and As by cellular sequestration, homeostasis of intracellular metal ions as well as adjustment of metal transport [153]. Apart from detoxification of heavy metals, plant MTs also play a role in maintenance of the redox level [137,154], repair of plasma membrane [137,155], cell proliferation and its growth, repair of damaged DNA [156] and scavenge ROS [156]. Mutualistic symbiotic association of *Arbuscular* mycorrhizal also occurs with roots of most vascular plant species under different climatic conditions in which they improve the mineral nutrition position of plants and augment their tolerance towards abiotic stresses and pollutants while benefiting from the photosynthetic assimilations supplied by the plants [137].

## 7. Role of Plant Growth-Promoting Bacteria (PGPR) in Plant Growth under Abiotic Stress

Understanding the mechanisms of PGPR in stimulating plant growth activities is critical for maintaining food production which subsequently improves food security. Most rhizospheric bacteria naturally tolerate environmental contaminants, hence, they are used in phytoremediation to remove organic pollutants and heavy metal contaminants in food crops. Rhizosperic bacteria make nutrients available in soils for plant growth, produce phytohormones such as Indole 3-acetic acid and also protect the plant against pathogens as well as remediating the soil of contaminants [157,158,159,160,161,162]. These PGPR live in the surrounding of the rhizosphere of the host plant where they boost plant growth and development by direct or indirect mechanisms [163]. Direct mechanisms include siderophore production, phosphate solubilization and 1-aminocyclopropane-1-carboxylate deaminase synthesis which enable the plant to withstand abiotic stress conditions by reducing ethylene levels, and enhances plant growth hormone production [69,161,163]; while the indirect mechanism of growth promotion involves the PGPR acting as biocontrol agents and detoxifying noxious substances such as heavy metals and pesticides [128,164]. The PGPRs associated with the rhizosphere could be extracellular plant growth promoting rhizobacteria (ePGPR) or intracelluar plant growth promoting rhizobacteria (iPGPR) depending on the level of interaction with the host plant root cells [165,166]. Most bacterial genera are ePGPR and include *Erwinia*, *Flavobacterium*, *Micrococcus*, *Pseudomonas*, *Serratia*, *Chromobacterium*, *Caulobacter*, *Azospirillium*, *Azotobacter* and *Agrobacterium* [163]. These PGPR produce various substances that enhance plant growth under abiotic stress. These include:

### 7.1. Siderophore Production

Rhizobacteria enhances plant growth by solubilizing usually weakly soluble nutrients with either bacteria siderophores or reducing the pH through secreting acidic organic compounds [161]. Siderophores are metal chelating agents with low molecular masses (200–2000 Da) that are formed by microorganisms and plants, especially under Fe-limiting conditions [167]. Iron is one of the essential nutrients required for plant metabolism but it is deficient in soil [168]. When Fe is limited in the soil, microbial siderophores solubilize and remove Fe from the soil and supply plants with Fe, thereby enhancing their growth [169]. Siderophores are also produced by plants; these are called phytosiderophores. The phytosiderophores are hexadentate ligands that synchronize Fe(III) with their amino and carboxyl groups [170] and range between 500 and 1000 Da [167]. Discharge of phytosiderophore to the rhizosphere causes chelatation of Fe from the soil by forming Fe(III)–phytosiderophore complexes that can be consequently transported across the root plasma membrane [167,171].

### 7.2. Phosphate Solubilization

Phosphorous is required by plants as a macronutrient but it reacts naturally with Fe, aluminum and Ca resulting in its precipitation and, hence, making it unavailable to plants. Plants are able to take up the little phosphorous available in the soil either as H_2_PO_4_^−^ (monobasic) or HPO_4_^2−^ (dibasic) ions [163]. Microorganisms capable of converting phosporous to a soluble form for plants are referred to as phosphate-solubilizing microorganisms (PSMs). Those that inhabit the rhizosphere of plants and supply phosphorous to the plant are called phosphate-solubilizing bacteria (PSB), some of which include *Azotobacter*, *Bacillus*, *Beijerinckia*, *Burkholderia*, *Enterobacter*, *Erwinia*, *Flavobacterium*, *Microbacterium*, *Pseudomonas* and *Rhizobium* and *Serratia* [163,172]. These phosphate-solubilizing bacteria supply phosphorous to the plant under stress conditions and augment plant growth by enhancing biological nitrogen (N_2_) fixation and making other trace elements accessible via plant-growth-promoting substances [91,161,172].

### 7.3. Aminoacyclopropane-1-Carboxylate Deaminase Production

Many PGPR directly stimulate growth in plants by synthesizing the enzyme 1-aminocyclopropane-1-carboxylate (ACC) deaminase which in harsh conditions such as drought, salinity, or heavy metal contamination, facilitate plant growth by cleaving and sequestering ACC thus reducing ethylene levels and making the plants capable of withstanding abiotic stress in the environment [173,174,175]. These PGPR with ACC deaminase activity, breakdown ACC to a-ketobutyrate and ammonia which sequentially decreases the quantity of ACC in the plants, thereby developing an extensive root system for the plant [172,176,177]. Hence, toxic levels of ethylene accumulation are prevented which will otherwise lead to plant death. The ACC-producing bacteria act as a sink for ethylene reduction. Most often, isolates with ACC deaminase activity have the ability to produce IAA and siderophores.

### 7.4. Indole-3-Acetic Acid Production

Eighty percent (80%) of microorganisms isolated from the rhizosphere of different crops have the capacity to produce and release auxins as secondary metabolites [161]. Indole-3-acetic acid (IAA) is important in plant–microbe relations especially between plants and rhizobacteria which stimulate plant growth through extensive root systems and protect the plant against abiotic stress [178]. Indole-3-acetic acid is synthesized by PGPR using tryptophan obtained from roots in the rhizospheric region. According to Spaepen and Vanderleyden [178], plant growth and development is regulated by exogenous levels of IAA since a low amount enhances root elongation and a high amount decreases primary root length, stimulate formation of lateral roots and increases root hair formation. The IAA molecules (after production) enter plant cells where they enhance plant growth or increase ethylene levels by activating ACC synthase activity. The IAA synthesized by rhizobacteria enhances root elongation and surface area, making soil nutrients more available and thereby enhancing root exudates which gives the rhizospheric bacteria more nutrients for their activity [161,164].

Some plant-growth-promoting rhizobacteria produce hydrogen cyanide which may be detrimental to the plant in high concentrations. Consequently, it is essential that the right strain or isolate of rhizobacteria be identifed and used to enhance plant growth under abiotic stress conditions.

## 8. Bioremediation Using Advanced Molecular Techniques and Genetically Engineered Organisms

Microorganisms are utilized in bioremediation because of their ability to degrade environmental pollutants due to their metabolism via biochemical pathways related to the organisms activity and growth. Through the process of co-metabolism, microorganisms are able to degrade to harmless end products hazardous substances found in polluted environments [179]. The cleanup of polluted environments using indigenous microorganisms has not yielded much positive results. For example, indigenous bacteria cannot remove heavy metals such as Hg from the environment. However, recombinant DNA technology has a major role to play in bioremediation of heavy metal contamination as it enhances the remediation process. The advent of recombinant DNA technology in the 1970s with the discovery of restriction enzymes and DNA ligases made it possible to alter the genome of living organisms. Since then, the metabolic potentials of microorganisms have been studied and microbes genetically modified for specific purposes. The aim of genetic engineering for bioremediation is to modify plants, microorganisms and enzymes so that they would be useful tools for degradation of harmful substances [180]. Genetic engineering has made it possible to engineer bacteria for the removal of heavy metals such as As, Cd, Cu, Fe, Hg and Ni [181,182,183]. However, the rate of degradation depends on the catalytic efficiency of enzymes residing in the cells or those induced to particular substrate [184].

Genetically engineered microorganisms (GEMs) contain foreign genes inserted into their genome from another organism of the same or different species using recombinant DNA technology. These engineered microbes have been used to obtain competent strains for bioremediation of contaminated environment by possessing enhanced ability to breakdown a variety of contaminants [3]. Reports have been made on the use of genetically engineered *Eschericia coli* strain M109 and *Pseudomonas putida* containing the *mer*A gene to effectively eradicate Hg from contaminated soils and sediments [185,186,187]. Azad et al. [183] gave a comprehensive review of the use of genetically engineered bacteria and plants in the bioremediation of sites contaminated with heavy metals and other organic pollutants. Most of the techniques used involved identification and insertion of genes involved in metal uptake into competent bacterial cells and plants. Genes that have been widely used include the *mer*A gene for Hg up take, phenol catabolic genes (*pheA*, *pheB*, *pheC*, *pheD* and *ph*eR) [188] and the *Ars*M gene for the removal of As from contaminated soils [189]. In a recent review by Dixit et al. [3], addition of *mer* operon from *Escherichia coli* which codes for Hg^2+^ reduction into genetically engineered bacterium *Deinococcus geothemalis* gave the microorganism the ability to reduce Hg contamination at high temperatures by expression of the *mer* genes. Similarly, *Cupriavidus metallidurans* strain MSR33 genetically engineered with a pTP6 plasmid that provided genes (*merB* and *merG*) regulating Hg biodegradation along with the synthesis of organomercurial lyase protein (*merB*) and mercuric reductase (*merA*) also gave the microorganism the ability to reduce Hg contamination from polluted sites [3,190]. *Pseudomonas* strains have also been made resistant to Hg due to the introduction of novel genes into the strain using pMR68 plasmid [191]. The n-alkane-degrading microorganisms possess specific genes such as *alkB*, *alkB1*, *alkB2*, *alkM*, aromatic hydrocarbons: *xylE*, and polycyclic aromatic hydrocarbons: *ndoB*, *nidA*, most often located on plasmids to enable horizontal gene transfer and used as markers for the identification of microbial biodegradation [180]. The utilization of GEMs to facilitate the remediation process is essential in the war against toxic substances in the environment. In order to use GEMs for bioremediation, it is essential that the stability of the microbes be maintained before their field application as the catabolic activity of released GEM is linked primarily with the stability of the recombinant plasmid introduced into the organism [192,193].

New metabolic pathways are made which enable the engineered bacteria to convert toxic types of heavy metals to less toxic or innocuous forms thereby enhancing the bioremediative processes. The most often used techniques include constructing new pathways and replacement of existing gene sequences as well as introduction of single genes or operons into the microorganism [179]. Microorganisms and their enzymes have been genetically modified to degrade organic pollutants in the environment using recent techniques such as site directed mutagenesis and rational designing [194]. New recombinant DNA techniques used to engineer pollutant-degrading microorganisms include the use of new vectors to introduce new gene fragments into a potential host, developing new methods for regulating gene expression and the use of targeted and random mutagenesis which increases the activity of biodegrading enzymes [180,195].

Microbial biosensors are currently being used to establish the amount of pollutants in contaminated sites quickly and precisely and are developed using genetic engineering. Dixit et al. [3] reported the use of biosensors to estimate the levels of heavy metals such as Cd, Ni, Hg, Cu and As in contaminated sites. However, the use of biosensors is limited due to variation in response times, detection thresholds, sensitivity, signal relaxation lengths, as well as stability [194]. Utilization of genetic engineering ensures greater opportunities for obtaining effective pollutant-degrading microorganisms as they could possess higher potential of environmental cleanup than the indigenous microbes. In order to successfully use GEMs for bioremediation in harsh environmental conditions, it is essential to preserve the recombinant bacterial population in the soil, with suitable environmental conditions prepared and the recombinant bacteria should be able to withstand opposition from indigenous bacterial populations [3]. Therefore, other novel molecular techniques should be explored to screen and isolate microorganisms for use in heavy metal bioremediation.

Advancements of modern techniques in genetics and omics such as genomics, proteomics and metabolomics have enabled scientist to study catabolism of organic pollutants by microorganisms which has made it possible to understand the physiology, ecology and biochemistry of polycyclic aromatic hydrocarbon (PAH)-degrading microorganisms [193]. It is now possible to study in detail the physiology of microorganisms connected with elimination of contaminants from the environment using whole genome sequencing [193,196]. Characterization of genes encoding bacterial inorganic transformation have paved the way for using molecular genetics to enhance metal tolerance [38]. Using gene technology, transgenic plants can be obtained with enhanced bioremediation abilities. This is done by insertion or over expression of specific genes in the DNA of the plant which is an effective way of increasing the capacity of plants for phytoremediation. It also involves using molecular mechanisms of detoxification via genetic engineering to confer on such plants the ability to effectively metabolize pollutants and degrade xenobiotics. Areas with metal contamination can be effectively remediated by genetically engineering endophytes and PGPR for degeneration of soil pollutants [3,197]. When genes determining antioxidant enzymes, or enzymes involved in the biosynthesis of glutathione and other phytochelatins are over-expressed, this enhances metal accumulation capacity and tolerance [38,198]. The report of Mani and Kumar [38] showed that few plant species expressing modified *merA* genes, such as rice and tobacco are resistant to at least ten times more concentrations of Hg than those that kill non-transgenic control [38]. Transgenic plants, such as *Arabidopsis thaliana*, *Nicotiana tabaccum*, *Brassica juncea*, *Brassica oleraceae var botrytis* and *Lycopersicon esculentum*, have been used for bioremediation of pollutants [185]. Reports have been made on production of transgenic plants with the ability to reduce ethylene levels by expressing ACC deaminase genes [199], remove and transport Hg from the soil to the shoot by expressing *merA* and *merB* genes, as well as degradation of pollutants through insertion of xenobiotic degradation genes into their root systems. Research on other fast-growing plants with metal accumulation ability should be promoted and more genera of microorganisms identified for genetic improvement of plants and rhizospheric microorganisms for phytoremediation. Aside from the *merA* genes, other genes should be studied for possible use for remediation of other heavy metals.

DNA microarrays, a high-throughput technique that identifies several genes in a single test, has overcome the shortcoming of other culture-independent approaches. GeoChip array is the most widespread gene array technique used for studying the function of genes. It targets numerous genes that participate in various geochemical cycles of carbon, nitrogen, phosphorus and sulfur, as well as metal resistance and reduction and degradation of pollutants [200].

Care must be taken in the introduction of genetically engineered microbes into the environement for bioremediation as it is possible for horzintal gene transfer to occur between the engineered microbes and natural microbes in the environment. Environmentalists are thoughtful of horizontal gene transfer of the engineered microorganisms with the indigenous microbes. This is because the microbes are capable of spreading rapidly in the environment and transfer resistant genes from one microbe to another via plasmids which enable them to adapt to new ecological environments. The effects such microbes will have on the indigenous microbes in the environment would need to be studied before release to contaminated sites. Nevertheless, for any microbial-based technology encircling bioremediation process to be adopted, it is crucial to monitor implanted recombinant strains of bacteria and design strategies to program cell death once the biocatalyst had completed its task, or in the event genetically modified genes get accidentally transferred [36]. Suicidal gene systems should therefore be developed in this regard so that horizontal gene transfer would not occur when engineered microbes have completed the remediation of contaminated sites. Horizontal gene transfer can also be prevented by the use of anti-sense technology which involves inserting antisense RNA-regulated plasmids and protein plasmids into the microbe which terminate or degrade after carrying out their work in remediation [183,201]. There is always delay in introducing GEMs into the environment for bioremediation because of some safety and legal issues and the public perception of the risks of GEMs [194]. This has resulted in rigid regulation by various biosafety regulatory bodies such as the United Nations Environmental Programme (UNEP) and the United States Environmental Protection Agency (EPA) which supervise the regulation of genetically modified organisms (GMOs) and living modified organisms [194]. Recombinant DNA technology is essential for the bioremediation process as it enables researchers to analyze, monitor and assess the implementation of the process [102]. However it should be used with caution and in accordance with biosafety regulations.

## 9. Future Prospects for Bioremediation

Considering the importance of transgenic microbes in greatly enhancing detoxification and degradation of xenobiotics and heavy metal contaminants, more studies should be carried out to enhance their survival when released into the environment for bioremediation, beacuse their survivability is currently poor. Environmental factors such as temperature and low nutrient concentrations and other factors which are not easily contolled, can hamper their utilization and the effectiveness of the bioremediation process [104]. Although efforts have been made in trying to prevent horizontal gene transfer from engineered microbes to indigenous microorganisms, using anti-sense RNA and suicidal genes, the use of antibiotic genes as selectable markers should be discontinued and replaced with other selectable markers to avoid antibiotic resistance genes being unintentionally transferred to other soil microorganisms. Moreover, more research is required to fully understand the metabolic pathways of transgenic plants and microbes used in bioremediation so as to ascertain their effectivenss and possible side effects if used for bioremediation. Hyperaccumulator plants with high biomass production should be identified and enhanced through genetic engineering to effectively extract heavy metals from the environment through the process of phytoextraction, which has proven to be an effective phytoremediation process. The ability of the microorganisms used in bioremediation to compete with indignous microbial population is essential for the success of bioremmediation.

## 10. Conclusions

This review highlighted the effects of heavy metal contamination caused by some human activities on the environment, the possible health hazards, as well as the various mechanisms and enzymatic reactions used by plants and microbes to effectively remediate polluted environments. It revealed the usefulness of bioremediation as a better substitute for the removal of heavy metals from contaminated sites compared to the physico–chemical methods which are less efficient and expensive due to the amount of energy expended. Microorganisms and plants possess inherent biological mechanisms that enable them to survive under heavy metal stress and remove the metals from the environment. These microbes use various processes such as precipitaton, biosorption, enzymatic transformation of metals, complexation and phytoremedation techniques of which phytoextraction and phytostabilization have been very effective. However, the environmental conditions need to be adequate for effective bioremediation. The use of hyperaccumulator plants to remediate contaminated sites depends on the quantity of metal at that site and the type of soil. Environmental factors play a major role in the success of bioremediation as the microbes used will be hampered if appropriate environmental conditions are not available. More rapidly growing plants with high phytoextraction ability should be identified for the remediation of pollutants from soil. Moreover, assessment of metal stress on beneficial rhizospheric microorganisms and crops should be carried out and effective ways of enhancing the bioremediation process predicted. Transgenic microbes and plants could effectively remediate contaminated sites of heavy metal and organic pollutants but its use should be subject to stringent biosafety procedures to ensure that there is no health or environmental hazards. More efficient ways of using transgenic plants and microbes should be identified that will enable effective remediation of polluted environments without horzontal transfer of recombinant plasmids or pollens to indigenous organisms, which is currently a major challenge. Synthetic biology is an emerging technology that will be useful to synthesize microbial consortia with the ability to degrade and remove heavy metals from agricultural soils using the metabolic properties of such consortia of organisms. This technology should be promoted for more effective remediation of the environment from pollutants. Metagenomic approaches and metabolic analysis should also be used to study the functional composition of microbial communities within the polluted sites for metal resistance genes that could be used to improve specific heavy metal degradation strains of microbes. Public perception of the use of gene technology for bioremediation will also need to change for its effective utilization; this will require cooperation between researchers and environmentalist.

## Figures and Tables

**Table 1:** Microorganisms used in heavy metal remediation of contaminated sites.

Class of Microorganisms	Heavy Metal Removed	References
1. Bacteria		
*Bacillus cereus strain* XMCr-6	Cr (VI)	[72]
*Kocuria flava*	Cu	[61]
*Bacillus cereus*	Cr (VI)	[61,73]
*Sporosarcina ginsengisoli*	As (III)	[61,74]
*Pseudomonas veronii*	Cd, Zn, Cu	[61,75]
*Pseudomonas putida*	Cr (VI)	[76]
*Enterobacter cloacae* B2-DHA	Cr (VI)	[77]
*Bacillus subtilis*	Cr (VI)	[76]
2. Fungi		
*Aspergillus versicolor*	Ni, Cu	[61,78]
*Aspergillus fumigatus*	Pb	[79]
*Gloeophyllum sepiarium*	Cr (VI)	[80]
*Rhizopus oryzae* (MPRO)	Cr (VI)	[81]
3. Yeast		
*Sacharomyces cerevisiae*	Pb, Cd	[82,83]
4. Algae		
*Spirogyra* spp. and *Cladophora* spp.	Pb (II), Cu (II)	[61,84]
*Spirogyra* spp. and *Spirullina* spp.	Cr Cu, Fe, Mn, Zn	[61,85]
*Hydrodictylon*, *Oedogonium* and *Rhizoclonium* spp.	As	[60,61]

**Table 2:** Some hyperaccumulator plants used in phytoextraction of heavy metals.

Family	Species	Heavy Metals	References
Asteraceae	*Berkheya coddii*	Ni	[108]
Asteraceae	*Helianthus annuus*	Pb, Cd, Zn	[101,109]
Brassicaceae	*Alyssum bertolonii*	Ni	[110]
Brassicaceae	*Alyssum murale*	Ni	[111]
Brassicaceae	*Arabidopsis halleri*	Zn, Cd	[112]
Brassicaceae	*Arabidopsis halleri*	Cd Cd	[113]
Caryophyllaceae	*Minuartia verna*	Zn, Cd, Pb	[114]
Crassulaceae	*Sedum alfredii*	Pb	[7,115]
Euphorbiaceae	*Euphorbia cheiradenia*	Cu, Fe, Pb, Zn	[1]
Fabaceae	*Astragalus racemosus*	Se	[116]
Fabaceae	*Medicago sativa*	Pb	[7]
Poaceae	*Spartina argentinensis*	Cr	[117]
Pteridaceae	*Pteris vittata*	As	[118,119,120]
Pteridaceae	*Pteris vittata*	Hg	[121]
Violaceae	*Viola boashanensis*	Pb, Zn, Cd	[122]
